# Effect of nitrous oxide on cisatracurium infusion demands: a randomized controlled trial

**DOI:** 10.1186/1471-2253-10-14

**Published:** 2010-08-18

**Authors:** Hanna L Illman, Heikki MJ Antila, Klaus T Olkkola

**Affiliations:** 1 Department of Anesthesiology, Intensive Care, Emergency Care and Pain Medicine, University of Turku and Turku University Hospital, Turku, Finland

## Abstract

**Background:**

Recent studies have questioned our previous understanding on the effect of nitrous oxide on muscle relaxants, since nitrous oxide has been shown to potentiate the action of bolus doses of mivacurium, rocuronium and vecuronium. This study was aimed to investigate the possible effect of nitrous oxide on the infusion requirements of cisatracurium.

**Methods:**

70 ASA physical status I-III patients aged 18-75 years were enrolled in this randomized trial. The patients were undergoing elective surgery requiring general anesthesia with a duration of at least 90 minutes. Patients were randomized to receive propofol and remifentanil by target controlled infusion in combination with either a mixture of oxygen and nitrous oxide (Nitrous oxide/TIVA group) or oxygen in air (Air/TIVA group). A 0.1 mg/kg initial bolus of cisatracurium was administered before tracheal intubation, followed by a closed-loop computer controlled infusion of cisatracurium to produce and maintain a 90% neuromuscular block. Cumulative dose requirements of cisatracurium during the 90-min study period after bolus administration were measured and the asymptotic steady state rate of infusion to produce a constant 90% block was determined by applying nonlinear curve fitting to the data on the cumulative dose requirement during the study period.

**Results:**

Controller performance, i.e. the ability of the controller to maintain neuromuscular block constant at the setpoint and patient characteristics were similar in both groups. The administration of nitrous oxide did not affect cisatracurium infusion requirements. The mean steady-state rates of infusion were 0.072 +/- 0.018 and 0.066 +/- 0.017 mg * kg-1 * h-1 in Air/TIVA and Nitrous oxide/TIVA groups, respectively.

**Conclusions:**

Nitrous oxide does not affect the infusion requirements of cisatracurium.

**Trial registration:**

ClinicalTrials.gov NCT01152905; European Clinical Trials Database at http://eudract.emea.eu.int/2006-006037-41.

## Background

Volatile anesthetics are known to affect the pharmacodynamics of neuromuscular blocking agents [1-3]. For this reason neuromuscular blocking agents are usually studied under nitrous oxide anesthesia supplemented with opioids and intravenous hypnotics. Unlike volatile anesthetics, nitrous oxide has been thought to have no effect on the dose-response relation of neuromuscular blocking drugs.

However, recent studies have questioned our previous understanding on the effect of nitrous oxide on muscle relaxants since nitrous oxide has been shown to potentiate the action of bolus doses of mivacurium, rocuronium and vecuronium [4-6]. Whether this interaction is due to an alteration in pharmacokinetics or pharmacodynamics remains, however, completely unknown. Evidence from one study on the infusion requirements of rocuronium during nitrous oxide demonstrated that nitrous oxide does not affect rocuronium in a clinically significant degree [7]. Thus, different study methodology appears to give different results on the interaction between nitrous oxide and rocuronium.

Because the mechanism of action of nitrous oxide on muscle relaxants is unknown and because it is controversial whether there is an interaction between nitrous oxide and muscle relaxants, we found it important to repeat our previous study [7] using cisatracurium, a muscle relaxant with a different chemical structure and elimination kinetics. Cisatracurium, as opposed to the aminosteroid relaxant rocuronium, is a benzyl isoquinoline compound with a novel pharmacokinetic profile since its elimination kinetics is essentially independent of liver and renal function [8]. We used a closed-loop feedback control method of administering cisatracurium to maintain a constant neuromuscular block of 90%. The interaction between cisatracurium and nitrous oxide was measured by determining the infusion requirements to produce 90% neuromuscular block with cisatracurium.

## Methods

This study was performed in accordance with the Good Clinical Research Practice Guidelines for pharmacodynamics studies of neuromuscular blocking agents [9]. After obtaining approval by the ethics committee of the Hospital District of Southwest Finland to conduct the study, 70 patients providing informed written consent were enrolled. We used a randomized study design in parallel groups. Based upon previous studies [3] it was calculated that 35 patients would be required in each group to establish a 15% difference in cisatracurium requirements at a level of significance of *P *= 0.05 and a power of 80%. The patients were aged 18-75 years, their ASA physical status 1-3, and they were scheduled to undergo elective surgery requiring general anesthesia with a duration of at least 90 minutes. Patients with significant renal, hepatic or cardiac disease were excluded from the study, as were patients with raised intracranial pressure, a body mass index greater than 32.5 kg/m^2^, patients suffering from neurologic disease or receiving medication known to affect neuromuscular function.

The patients received oral premedication consisting of 3.75-7.5 mg midazolam approximately 1 hour prior to induction of anesthesia. All patients received total intravenous anesthesia (TIVA) using target controlled infusion of propofol and remifentanil. One group of patients received a mixture of air with 30% oxygen (Air/TIVA group) and the other group received nitrous oxide with 30% oxygen (Nitrous oxide/TIVA group). The patients were randomly assigned to one of these groups, each group consisting of 35 patients. The initial target of propofol was set at 4 μg/ml and if necessary adjusted to 6 μg/ml for adequate induction. After induction the target was maintained at 4 μg/ml until the end of surgery. The target of remifentanil was initially set at 2 ng/ml and later adjusted between 1.5-6 ng/ml, according to clinical needs. A decrease in systolic blood pressure below 85 mmHg or a decrease in mean blood pressure below 55 mmHg, respectively, was treated by decreasing the target of remifentanil to a minimum level of 1.5 ng/ml. In addition, the patients received rapid infusion of Ringer's acetate solution and/or 5-10 mg of intravenous ephedrine, when considered necessary. In hypertensive patients treatment of hypotension was initiated if a 30% decrease in blood pressure was detected. Fresh gas flow was kept at 10 l/min until tracheal intubation, using the above mentioned gas mixtures. During maintenance of anesthesia gas flow was set at 5 l/min, with the end-tidal nitrous oxide concentration kept above 65% in the patients receiving nitrous oxide.

The degree of neuromuscular blockade was assessed every 20 seconds, throughout the procedure, using a Datex Relaxograph^® ^monitor (Datex, Helsinki, Finland). Surface electrodes were attached over the ulnar nerve and over the first interosseus muscle and the index finger [10]. The train-of-four sequence was used (2 Hz frequency, 100 ms pulse width), the stimulus output being a rectangular wave with a current of 0-70 mA. The machine calibrated automatically by searching for the optimal signal levels before setting the supramaximal level. Control electromyographic values were obtained after induction and following this, a stable baseline calibration signal was awaited and a second calibration was performed approximately 10 minutes after induction of anesthesia. During this period patients were ventilated manually with a mask. The degree of neuromuscular blockade was defined as the ratio of the measurement of the first twitch in the train-of-four sequence to the corresponding control value.

After obtaining a stable calibration signal, a bolus dose of 0.1 mg/kg cisatracurium was administered. We used the ideal body weight (IBW), as defined by Devine's equation, for the calculation of the dose of cisatracurium [11]. Tracheal intubation was performed and the patients were mechanically ventilated using either of the above mentioned gas mixtures. Bolus administration of cisatracurium was followed by infusion of cisatracurium by a model-driven closed-loop feedback system as described previously [12]. An infusion pump (Fresenius Infusomat CP^®^; Bad Homburg, Germany) and the Relaxograph^® ^were attached to a Compaq^® ^portable 386 computer (Houston, TX) by means of a serial RS232C interface. The study time was 90 min for all patients. Propofol, remifentanil, and cisatracurium infusions were continued as long as clinically indicated, but only the initial 90-min period from the administration of the bolus dose of cisatracurium was analyzed. Palmar skin temperature was measured and kept above 33°C, and end-tidal carbon dioxide was maintained at 34-40 mm Hg (4.5-5.3%). Depth of anesthesia was monitored using the Bispectral Index.

The desired level of neuromuscular block (*i.e*., the set point) was set to 90% (the first twitch in the train-of-four sequence = 10% from control). Controller performance was measured by calculating the mean offset from set-point and the mean SD from set-point during feedback infusion as described previously. The measured values for effect and rate of the infusion were saved on the computer. The possible effect of nitrous oxide on the infusion requirements of cisatracurium was quantified by comparing the asymptotic steady state rates of infusion for 90% block between the groups. To estimate the asymptotic steady state rates of infusion, we used nonlinear curve fitting for the cumulative dose curve of cisatracurium during the 90-min study period [13].

$$\text{Cumulative dose of cisatracurium}=\text{D}\cdot (1-{\text{e}}^{-\text{kt}})+{\text{I}}_{\text{ss}}\text{t},$$

where D is the amount of cisatracurium in its apparent distribution volume, k is the relative rate of distribution of cisatracurium, I_ss _is the asymptotic steady state infusion rate of cisatracurium, and t is the duration of administration of cisatracurium. The asymptotic steady state rates of infusion were given as actual values and per kilogram ideal body weight (I_ss_/IBW). By the end of surgery, all patients received a neostigmine-glycopyrrolate mixture to reverse neuromuscular block according to our normal routine.

For statistical analysis, we used the Student *t *test and chi-square test. *P *< 0.05 was considered to indicate statistically significant differences between the two groups. All results are given as mean ± SD. For continuous variables, we also calculated 95% confidence intervals of the differences in mean values. All data were analyzed with use of the statistical program Systat for Windows, version 10.2 (Systat Software, Richmond, CA).

### Registration

ClinicalTrials.gov NCT01152905; European Clinical Trials Database at http://eudract.emea.eu.int/2006-006037-41

## Results

**Figure 1:**
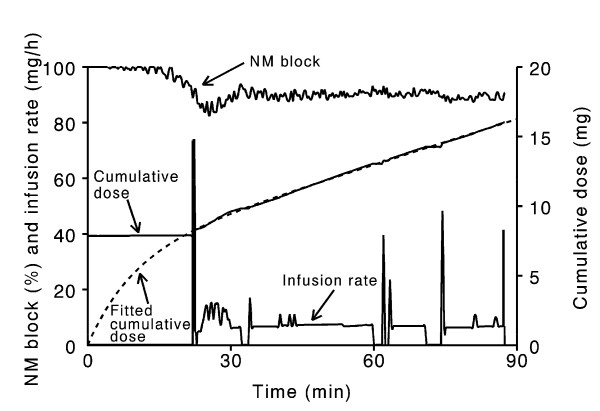
**Neuromuscular block and rate of infusion of cisatracurium**. Data for one representative patient in the Nitrous oxide/TIVA (total intravenous anesthesia) group showing the rate of infusion (I_ss_) necessary to produce a constant 90% neuromuscular (NM) block by closed-loop infusion of cisatracurium, the corresponding cumulative dose requirements of cisatracurium, the fitted cumulative dose requirements and the measured NM block.

**Figure 2:**
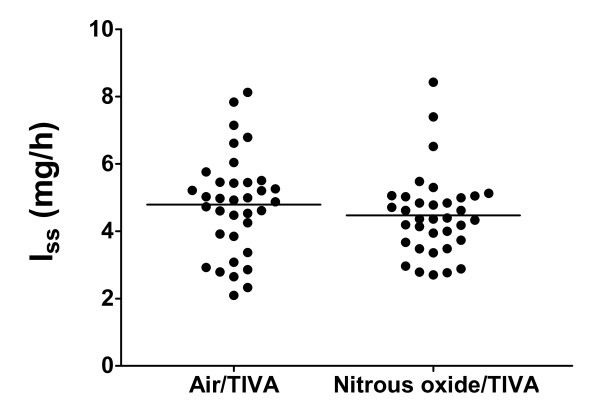
**Steady-state infusion rates**. Scattergram of the individual values of the rate of infusion (I_ss_) necessary to produce a constant 90% neuromuscular block during total intravenous anesthesia with air (Air/TIVA) and nitrous oxide (Nitrous oxide/TIVA). The horizontal line represents the mean value in each group. Cisatracurium was administered by model-driven closed-loop infusion.

Patient characteristics, controller performance and values for the cumulative dose of cisatracurium during the 90-min study period, I_ss _and I_ss_/IBW, are shown in Table 1. No statistically significant differences in patient characteristics or controller performance were observed in the two groups. The values for the average duration of infusion of cisatracurium in the two groups (55.6 ± 7.6 min in the Nitrous oxide/TIVA group and 58.5 ± 6.1 min in the Air/TIVA group) did not differ. Peripheral skin temperature, end-tidal carbon dioxide, average values for Bispectral Index and remifentanil consumption were also similar. Average Bispectral Index levels were 27.7 ± 6.3 in the Nitrous oxide/TIVA group and 30.2 ± 8.4 in the Air/TIVA group, respectively (*P *= 0.163), while the average cumulative doses of remifentanil were 524 ± 132 μg and 585 ± 163 μg, respectively (*P *= 0.091). There was a tendency for slightly more frequent ephedrine administration in the Nitrous oxide/TIVA group, but the difference was not statistically significant. Figure 1 shows an example of the time course of neuromuscular block and the cumulative dose requirements of cisatracurium for one representative patient in the Nitrous oxide/TIVA group. The cumulative dose of cisatracurium, I_ss _and I_ss_/IBW did not differ (Table 1 and Figure 2).

**Table 1:** Steady-state rate of infusion of cisatracurium controlled by closed-loop feedback system to maintain neuromuscular blockade constant at 90% during total intravenous anesthesia (TIVA) with air (Air/TIVA) or with nitrous oxide (Nitrous oxide/TIVA).

	Patients					Controller performance		Time to 10% recovery of T1 following the initial bolus (min)	Cumulative dose of cisatracurium/IBW (mg/kg)	Steady-state rate of infusion of cisatracurium	
				
Group	No. (M/F)	ASA (1/2/3)	Age (yr)	Weight (kg)	Height (cm)	Offset from set-point (%)	SD from set-point (%)			I_ss_ (mg/h)	I_ss_/IBW **(mg·kg**^**-1**^**·h**^**-1**^**)**
Air/TIVA	35 (20/15)	23/12/0	47.3 ± 12.6	75.0 ± 14.3	173 ± 11	0.96 ± 1.25	3.03 ± 1.34	31.5 ± 6.1	12.4 ± 2.6	4.8 ± 1.5	0.072 ± 0.018
Nitrous oxide/TIVA	35 (22/13)	18/16/1	48.6 ± 12.3	77.7 ± 15.1	174 ± 10	1.10 ± 1.28	2.95 ± 1.19	34.4 ± 7.6	12.1 ± 2.2	4.5 ± 1.2	0.066 ± 0.017
Mean difference (95% CI)			1.3 (-4.7, 7.2)	2.7 (-4.3, 9.7)	1.3 (-3.8, 6.3)	0.14 (-0.47, 0.74)	-0.08 (-0.68, 0.53)	2.9 (-0.4, 6.2)	0.3 (-1.5, 0.8)	-0.3 (-1.0, 0.3)	-0.005 (-0.014, 0.003)

Values are mean ± SD. There were no statistically significant differences between the groups.

ASA = American Society of Anesthesiologists physical status classification; CI = confidence interval of the difference in mean values; IBW = ideal body weight; I_ss _= asymptotic steady-state rate of infusion; I_ss_/IBW = asymptotic steady-state rate of infusion per kg ideal body weight; NMB = neuromuscular blockade; SD = standard deviation; T1 = first twitch in the train-of-four sequence.

## Discussion

The computerized closed-loop feedback infusion of cisatracurium kept the level of neuromuscular block at a reasonably constant level and thus allowed the accurate quantitation of the possible interaction of nitrous oxide with cisatracurium by assessing cisatracurium infusion requirements. Unlike observed earlier with bolus administration of mivacurium, rocuronium and vecuronium [4-6] but in good agreement with our own study with continuous infusion of rocuronium, nitrous oxide had no statistically significant effect on cisatracurium although the study was adequately powered to observe as small as 15% difference in infusion requirements at a level of significance of *P *= 0.05 and a power of 80%.

Our studies differ from previous studies on the interaction between nitrous oxide and muscle relaxants in many ways. Like our former study on rocuronium [7] but unlike previous studies using bolus techniques, we used a closed-loop feedback control method of administering cisatracurium to produce and maintain a relatively constant neuromuscular block of 90%. It was thus possible to quantitate any interaction between cisatracurium and nitrous oxide during maintenance of anesthesia with longer exposure to nitrous oxide and with minimal disturbance of the clinical routine. It has been suggested that propofol may affect the potency of neuromuscular blocking drugs. In one study a 20-min infusion of propofol caused a 50% rise in the potency of mivacurium as compared to a 5-min propofol infusion [14]. We used a target controlled infusion of propofol and the target was kept unchanged at 4 μg/ml in each patient during the maintenance of anesthesia for the entire study period. Because remifentanil is not known to affect the level of neuromuscular blockade [15] and because its cumulative dose during the 90-min study period and BIS-levels were similar in both groups, we believe that we were able to quantitate the effect of nitrous oxide on cisatracurium.

If the possible interaction of nitrous oxide and muscle relaxants is studied using bolus techniques, it has several implications. The study group of Kopman et al. [5] used the single-dose technique for the quantition of the nitrous oxide-rocuronium interaction. They estimated the value of the mean effective dose 50% (ED_50_) assuming that the dose-effect relation of rocuronium has a constant slope of 4.5 in a log-dose/logit plot. A 20% decrease of the mean ED_50 _was observed. Although this technique has weaknesses, it provides a rather robust estimate of the ED_50_, but with wide confidence intervals. If the true value of the slope were, for instance, 3.5 the estimate for the ED_50 _would increase by approximately 3%. The value of 5.5 would decrease ED_50 _by 1%. The single-dose technique can be used to estimate ED_95_, too. However, such calculations are seldom done because they are much more sensitive for having the correct value of the log-dose/logit slope. However, one might question the usefulness of ED_50 _for clinical purposes where normally at least 90% neuromuscular block is required for adequate surgical relaxation. We thus believe that it is more relevant to study the possible interaction of nitrous oxide and muscle relaxants using constant infusion of the muscle relaxant under investigation.

Other previous studies using bolus techniques in the assessment of the pharmacodynamics have yielded similar results as compared to Kopman et al. [5] Nitrous oxide has been shown to slightly affect the potency of both vecuronium [6] and mivacurium [4]. However, the duration of nitrous oxide administration before muscle relaxant was only 15 min in these rocuronium and mivacurium studies and 5 min in the vecuronium study [4-6]. While the interaction between muscle relaxants and volatile anesthetics is clearly a pharmacodynamic one, the mechanism of action of nitrous oxide on neuromuscular blocking drugs is still unknown. Volatile anesthetics do not seem to affect the pharmacokinetics of muscle relaxants, and it is generally assumed that nitrous oxide has no effect on the pharmacokinetics of muscle relaxants [16-20]. It has been proposed that nitrous oxide affects the neuromuscular junction directly and independently of its rate of accumulation in the muscle [4] or by altering the transfer of muscle relaxants to the site of action [5]. In fact the saturation of muscle tissue with nitrous oxide is less than 30% complete after 15 min of nitrous oxide anesthesia thus supporting the idea of an accumulation-independent effect of nitrous oxide on neuromuscular junction [4].

The reason for the disagreement between both our studies using continuous infusion and previous studies using bolus administration of muscle relaxants [4-6] is not at all clear. Obviously, the results of our studies cannot be directly compared to the previous studies, due to this difference in modes of administration. Ideally, the effect of anesthesia on the pharmacodynamics on both bolus dosage and continuous infusion should have been investigated consecutively in all our patients. However, this would have been time consuming and logistically impossible to carry out in our clinical setting.

It is logical to assume that the duration of the exposure to nitrous oxide has an effect but there is no definitive evidence. The more likely explanation is that nitrous oxide has only a minor effect, if any, on the neuromuscular action of muscle relaxants. While looking at the scattergram of the individual I_ss _values in the current study (Figure 2), it is plausible to conclude that the effect of nitrous oxide on cisatracurium pharmacodynamics is negligible, as was the case in our rocuronium study, and has no clinical significance. The same information is provided also by the 95% confidence intervals of the differences in mean I_ss _and I_ss_/IBW values. We conclude that nitrous oxide does not affect the infusion requirements of cisatracurium to a clinically significant degree.

## Conclusions

Nitrous oxide does not affect cisatracurium demand when using closed-loop computerized infusion of the muscle relaxant with the aim of maintaining a stable 90% neuromuscular block.

## Competing interests

M.D., Ph.D. Klaus Olkkola is a member of the advisory board of Finnish MSD Inc. Hanna Illman has received lecture fees from Finnish MSD Inc. and MSD Inc.

## Authors' contributions

All authors have made significant contributions to the design and conduct of this study, to the analysis and interpretation of the obtained data and to the preparation of this manuscript. All authors have read and approved the final manuscript.

## Pre-publication history

The pre-publication history for this paper can be accessed here:

http://www.biomedcentral.com/1471-2253/10/14/prepub
